# Predicting mortality risk in hospitalized COVID-19 patients: an early model utilizing clinical symptoms

**DOI:** 10.1186/s12890-023-02838-1

**Published:** 2024-01-10

**Authors:** Cong Nguyen Hai, Thanh Bui Duc, The Nguyen Minh, Lich Ngo Quang, Son Luong Cao Tung, Loi Trinh Duc, Sy Duong-Quy

**Affiliations:** 1 Department of Tuberculosis and Respiratory Pathology, Military Hospital 175, Ho Chi Minh City, Vietnam; 2 Military Hospital 175, Ho Chi Minh City, Vietnam; 3 Clinical Research Unit, Lam Dong Medical College and Bio-Medical Research Centre, Dalat City, Vietnam; 4 Immuno-Allergology Division, Hershey Medical Center, Penn State College of Medicine, Hershey, Pennsylvania USA

**Keywords:** COVID-19, Mortality prediction, Clinical symptoms, MH175 scale

## Abstract

**Background:**

Despite global efforts to control the COVID-19 pandemic, the emergence of new viral strains continues to pose a significant threat. Accurate patient stratification, optimized resource allocation, and appropriate treatment are crucial in managing COVID-19 cases. To address this, a simple and accurate prognostic tool capable of rapidly identifying individuals at high risk of mortality is urgently needed. Early prognosis facilitates predicting treatment outcomes and enables effective patient management. The aim of this study was to develop an early predictive model for assessing mortality risk in hospitalized COVID-19 patients, utilizing baseline clinical factors.

**Methods:**

We conducted a descriptive cross-sectional study involving a cohort of 375 COVID-19 patients admitted and treated at the COVID-19 Patient Treatment Center in Military Hospital 175 from October 2021 to December 2022.

**Results:**

Among the 375 patients, 246 and 129 patients were categorized into the survival and mortality groups, respectively. Our findings revealed six clinical factors that demonstrated independent predictive value for mortality in COVID-19 patients. These factors included age greater than 50 years, presence of multiple underlying diseases, dyspnea, acute confusion, saturation of peripheral oxygen below 94%, and oxygen demand exceeding 5 L per minute. We integrated these factors to develop the Military Hospital 175 scale (MH175), a prognostic scale demonstrating significant discriminatory ability with an area under the curve (AUC) of 0.87. The optimal cutoff value for predicting mortality risk using the MH175 score was determined to be ≥ 3 points, resulting in a sensitivity of 96.1%, specificity of 63.4%, positive predictive value of 58%, and negative predictive value of 96.9%.

**Conclusions:**

The MH175 scale demonstrated a robust predictive capacity for assessing mortality risk in patients with COVID-19. Implementation of the MH175 scale in clinical settings can aid in patient stratification and facilitate the application of appropriate treatment strategies, ultimately reducing the risk of death. Therefore, the utilization of the MH175 scale holds significant potential to improve clinical outcomes in COVID-19 patients.

**Trial registration:**

An independent ethics committee approved the study (Research Ethics Committee of Military Hospital 175 (No. 3598GCN-HDDD; date: October 8, 2021), which was performed in accordance with the Declaration of Helsinki, Guidelines for Good Clinical Practice.

## Background

According to the World Health Organization (WHO), as of April 2023, there have been over 762 million confirmed cases of COVID-19 globally, resulting in more than 6.8 million deaths. In Vietnam, the WHO reported 11,527,326 confirmed cases of COVID-19 and 43,186 deaths [1]. 

Despite global efforts to control the COVID-19 pandemic, the emergence of new viral strains poses a continued threat. The number of cases remains high in many countries worldwide, and the global mortality rate for COVID-19 was estimated at 3.4%, surpassing previous estimates of approximately 2% [2]. Individuals infected with the delta variant of COVID-19 typically exhibited nonspecific clinical presentations, ranging from asymptomatic SARS-CoV-2 carriers to middle-to-moderate and critical COVID-19 cases, and in some instances, led to fatalities possibly due to COVID-19 [3, 4]. Symptoms spanned from mild fever, fatigue, cough, and shortness of breath to severe cases involving lower respiratory tract infections and pneumonia. A subset of these cases progressed to Severe Acute Respiratory Distress Syndrome (ARDS) or multiorgan failure, ultimately resulting in mortality. To effectively stratify patients, optimize resource allocation, and provide appropriate treatment and care, there is a pressing need for a simple and accurate prognostic tool capable of rapidly identifying individuals at high risk of mortality. Early prognosis is important because it enables the prediction of treatment outcomes and facilitates appropriate patient management. 

This study aimed to develop an early and straightforward prognostic model for predicting mortality among COVID-19 patients. Given the ongoing pandemic situation leading to a surge in patient numbers across medical facilities and considering the existing limitations in both medical equipment and the professional capacity of healthcare staff, especially in primary medical facilities in Vietnam. We opted for a research model centered on easily identifiable and assessable clinical symptoms. This choice diverges from prognostic models that incorporate numerous clinical and paraclinical variables, which can be challenging to implement. The model was based on initial and fundamental clinical information, which can be easily employed in various medical facilities and is suitable for the current clinical pandemic environment in Vietnam. Additionally, we aimed to explore the applicability of this model in early prognosis for patients with other acute respiratory infections caused by different viral etiologies, such as influenza A, Rhino, and Adeno, as these diseases share certain similarities in terms of pathogenesis and microbiology. The successful implementation of this model could contribute to the adoption of appropriate treatment approaches and ultimately reduce the mortality rate among affected patients.

## Methods

### Study design

Cross-sectional cohort study using convenience sampling. Epidemiological, demographic, clinical, and therapeutic information at the time of admission was collected using one uniform medical record design.

### Patient selection

 A total of 375 individuals aged 18 and above who were diagnosed with moderate to severe COVID-19 and were admitted to Military Hospital 175 were selected for this study. The enrollment period spanned from October 2021 to December 2022, and the selection criteria were based on the diagnostic and treatment guidelines outlined by the Vietnamese Ministry of Health [5]. 

### Moderate COVID-19

Saturation of Peripheral Oxygen (SpO2): 94–96%; Breathing rate: 20–25 times/minute; Lung damage on X-ray: < 50%; Also applies to mild cases with underlying medical conditions.

Severe COVID-19: SpO2: < 94%; Respiratory rate: > 25 times/minute; Lung damage on X-ray: > 50%.

**Table:** 

The principles of treatment for COVID-19 patients [5]		
	Moderate COVID-19	Severe COVID-19
Favipiravir	Yes	Yes
Remdesivir	Yes	Yes
Casirivimab 600 mg + Imdevimab 600 mg	Yes	No
Bamlanivimab + Etesevimab	Yes	No
Sotrovimab	Yes	No
Corticoid	Yes	Yes
Anticoagulants	Enhanced prophylactic dose	Treatment dose
Respiratory support	Oxygen nasal cannula, simple mask	High-Flow Nasal Cannula/Non-Invasive Ventilation, or bag mask ventilation
Antibiotics	Consideration	Yes
Hemodialysis	No	Remove cytokines for 3—5 days
ECMO	No	No
Treatment of underlying disease	Yes	Yes
Nutritional support, physical therapy and psychotherapy	Yes	Yes

Participants were excluded from the study if they had incomplete information, remained hospitalized for more than 30 days after admission, or had comorbidities such as active pulmonary tuberculosis, cancer, or preexisting consciousness disorders unrelated to COVID-19. The study protocol is approved by the Research Ethics Committee of Military Hospital 175 in biomedical research. 

### Data acquisition

Epidemiological, demographic, clinical, and therapeutic data were obtained upon admission using a standardized medical record format. Subsequently, patients were monitored to assess their outcomes within a 30-day period following admission. Based on these outcomes, the participants were categorized into two groups: the Mortality group, which consisted of individuals who succumbed to the illness, and the Survival group, which consisted of individuals who achieved successful recuperation. 

### Evaluation criteria

Confirmation of Severe Acute Respiratory Syndrome Coronavirus-2 (SARS-CoV-2) infection was made by using the Reverse Transcription Polymerase Chain Reaction (RT‒PCR) technique, following established protocols, in COVID-19 laboratories at Military Hospital 175. The criteria for patient discharge encompassed clinical stability, amelioration of respiratory symptoms, and the absence of SARS-CoV-2 RNA in at least two consecutive nasopharyngeal swab specimens collected at intervals of ≥ 24 h [3]. Patients who were diagnosed with two or more chronic underlying diseases before contracting COVID-19 are categorized as having multiple comorbidities. Acute confusion was characterized by impaired orientation, diminished attention, and aberrant perception. Oxygen demand was the requirement for oxygen support to maintain a patient's SpO2 at or above 94%. 

### Statistical analysis

The data were subjected to medical statistical methods and analyzed using SPSS 20.0 software. Categorical variables were compared using a Chi-square test, while quantitative variables were compared using Student's t test for two independent samples. Furthermore, ANOVA was employed to compare means among three or more samples. Risk factors for mortality were identified using univariable and multivariable logistic regression analyses. Discriminatory power was assessed using the area under the receiver operating characteristic (ROC) curve (AUROC). An AUROC value ranging from 0.8 to 0.9 is indicative of good discriminative ability. Additionally, the predictive value of the scale for mortality was determined based on measures such as sensitivity, specificity, positive predictive value, and negative predictive value. The *p*-value is derived from statistical tests, where *p* < 0.05 is deemed statistically significant. All confidence intervals reported in this study are two-sided 95% confidence intervals.

## Results

### Baseline characteristics of COVID-19 patients

According to the results of univariate logistic regression analysis, several characteristics were identified as strong risk factors associated with mortality. These factors included advanced age, preadmission requirement for oxygen treatment, presence of diabetes, hypertension, or chronic kidney disease, history of stroke, and presence of multiple comorbidities (Table 1).

 Additionally, independent risk factors for mortality in hospitalized COVID-19 patients were found to include six clinical variables: shortness of breath, a respiratory rate exceeding 25 breaths per minute, acute disorders of consciousness, SpO2 levels below 94%, a need for supplemental oxygen, and oxygen flow exceeding 5 L per minute (Table 2). Furthermore, the Mortality group exhibited significantly higher levels of white blood cells, neutrophils, D-dimer, creatinine, and C-reactive protein in the blood, as well as a higher incidence of pneumonia, while the platelet count was significantly lower compared to the Survival group. (Table 3).

**Table 1:** Baseline demographics and characteristics of the COVID-19 patients

Characteristics		Survival (*n* = 246)		Mortality (*n* = 129)		Total	*P* value
		*n*	%	*n*	%		
Gender	Male	110	44.7	56	43.4	166 (44.3%)	0.8
	Female	136	55.3	73	56.6	209 (55.7%)	
Age, years	> 50	136	55.3	120	93.0	256 (68.3%)	< 0.001
	$\overline{{\text{X}} }$± SD	52.8 ± 15.4		68.9 ± 12.4		59.4 ± 16.3	< 0.001
Duration of illness, days, $\overline{{\text{X}} }$± SD		6.1 ± 4.3		6.6 ± 4.1		6.3 ± 4.3	0.28
Prehospital oxygen, yes		07	2.8	38	29.5	45 (12%)	< 0.001
Diabetes mellitus, yes		41	16.7	62	48.1	103 (27.5%)	< 0.001
Hypertension, yes		100	40.7	101	78.3	201 (53.6%)	< 0.001
Chronic kidney disease, yes		01	0.4	09	07	10 (2.7%)	< 0.001
Chronic pulmonary disorders, yes		05	02	03	2.3	08 (2.1%)	0.85
Patients with any history of stroke, yes		05	02	14	10.9	19 (5.1%)	< 0.001
Multiple comorbidities, yes		41	16.7	69	53.5	110 (29.3%)	< 0.001

**Table 2:** Clinical characteristics of COVID-19 patients on admission

Characteristics		Survival (*n* = 246)		Mortality (*n* = 129)		Total	*p* value
		*n*	%	*n*	%		
BMI, kg/m^2^	Overweigh, yes	52	22.2	34	26.4	86 (23.7%)	0.37
	$\overline{{\text{X}} }$± SD	23 ± 3.7		22.8 ± 4.2		22.9 ± 3.9	0.68
Fever, yes		141	57.3	52	40.3	193 (51.5%)	0.002
Cough, yes		203	82.5	103	79.8	306 (81.6%)	0.52
Shortness of breath, yes		171	69.5	120	93.0	291 (77.6%)	< 0.001
Acute disorder of consciousness, yes		01	0.4	10	7.8	11 (2.9%)	< 0.001
Respiratory rate (breaths/minute)	> 25	25	10.2	35	27.1	60 (16%)	< 0.001
	$\overline{{\text{X}} }$± SD	20.8 ± 3.4		23.5 ± 8.3		21.7 ± 5.8	< 0.001
Pulse (beats/minute)	> 100	42	17.1	31	24.0	73 (19.5%)	0.1
	$\overline{{\text{X}} }$± SD	90.4 ± 15.2		91 ± 15		90.6 ± 15.1	0.7
SpO2 (%)	< 94	56	22.8	69.0	53.5	125 (33.3%)	< 0.001
	$\overline{{\text{X}} }$± SD	93.7 ± 10.4		91 ± 7.6		92.8 ± 9.6	0.005
Supplemental oxygen requirement, yes		150	61.0	114	88.4	264 (70.4%)	< 0.001
Flow of oxygen > 5 (liters/minute)		104	42.3	110	85.3	214 (57.1%)	< 0.001

**Table 3:** Baseline blood and biochemical indices of the COVID-19 patients

Characteristics	Survival	Mortality	*p* value
White blood cells, G/L, $\overline{{\text{X}} }$± SD	8.9 ± 7.1	10.7 ± 6.5	0.01
Platelets, G/L, $\overline{{\text{X}} }$± SD	239.5 ± 106.7	200.8 ± 93.6	0.001
Neutrophils, %, $\overline{{\text{X}} }$ ± SD	74.9 ± 13.6	84.1 ± 15.1	< 0.001
D-dimer, ng/ml, $\overline{{\text{X}} }$± SD	1619.8 ± 6480.2	5237.8 ± 14542.7	0.008
Ure, mmol/l, $\overline{{\text{X}} }$± SD	5.6 ± 4	9.8 ± 7.8	< 0.001
Creatinine, µmol/l, $\overline{{\text{X}} }$± SD	79.3 ± 21.7	111.9 ± 93.7	< 0.001
CRP, ng/ml, $\overline{{\text{X}} }$± SD	52.3 ± 61.8	100.2 ± 73.5	< 0.001
PCT, pg/ml, $\overline{{\text{X}} }$± SD	0.54 ± 2.3	0.68 ± 1.2	0.66
IL-6, pg/ml, $\overline{{\text{X}} }$± SD	116.4 ± 187.8	182.9 ± 311.2	0.24
Pneumonia, yes, *n* (%)	222 (90.2%)	126 (97.7%)	0.008

### Independent clinical risk factors for mortality predictive in hospitalized COVID-19 patients

We conducted a multivariate analysis using selected objective clinical symptoms that were easily assessable to identify factors with independent predictive value for mortality. The results of the multivariable regression analysis demonstrated that certain factors, including age over 50, presence of multiple comorbidities, shortness of breath, acute disorders of consciousness, SpO2 levels below 94%, and oxygen demand exceeding 5 L per minute, independently contributed to the prognosis of mortality (Table 4).

**Table 4:** Clinical risk factors for mortality in COVID-19 patients

Factors	OR (95% CI)	*p* value
Age > 50	6.4 (2.8, 14.4)	< 0.001
Multiple comorbidities	5.2 (2.7, 9.8)	< 0.001
Shortness of breath	3.7 (1.4, 9.7)	0.008
Consciousness disorders	24.6 (1.6, 383)	0.02
Respiratory rate > 25 breaths/minute	1.1 (0.5, 2.4)	0.8
SpO2 level < 94%	2.1 (1.1, 3.9)	0.02
The flow of oxygen > 5 L/minute	0.1 (0.07, 0.3)	< 0.001

### MH175 scale: a novel clinical scoring model for the prediction of mortality of hospitalized COVID-19 patients

**Fig. 1:**
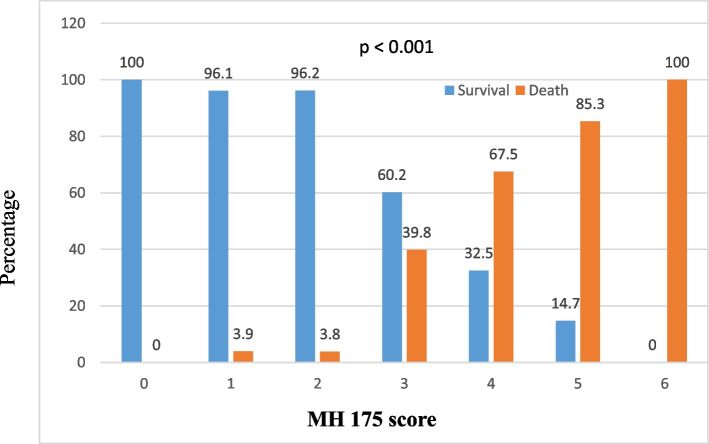
Mortality by the MH 175 score

Multivariate logistic regression analysis revealed that six clinical risk factors, including age over 50, presence of multiple comorbidities, shortness of breath, acute disorders of consciousness, SpO2 levels below 94%, and oxygen demand exceeding 5 L per minute, independently contributed to the risk of mortality in COVID-19 patients. Therefore, we developed a prognostic scale known as Military Hospital 175 (MH175) that incorporated each of these factors. Each risk factor was assigned a score of 1, resulting in a total score ranging from 0 to 6, with a score of 0 indicating low mortality and a score of 6 indicating high mortality. A score of 3 on the MH175 scale corresponded with a mortality rate of 39.8%, while a score of 4 corresponded with a mortality rate of 67.5%. The mortality rate increased to 85.3% for patients with a score of 5, and those with a maximum score of 6 points on the MH175 scale faced a mortality rate of 100% (Fig. 1).

**Fig. 2:**
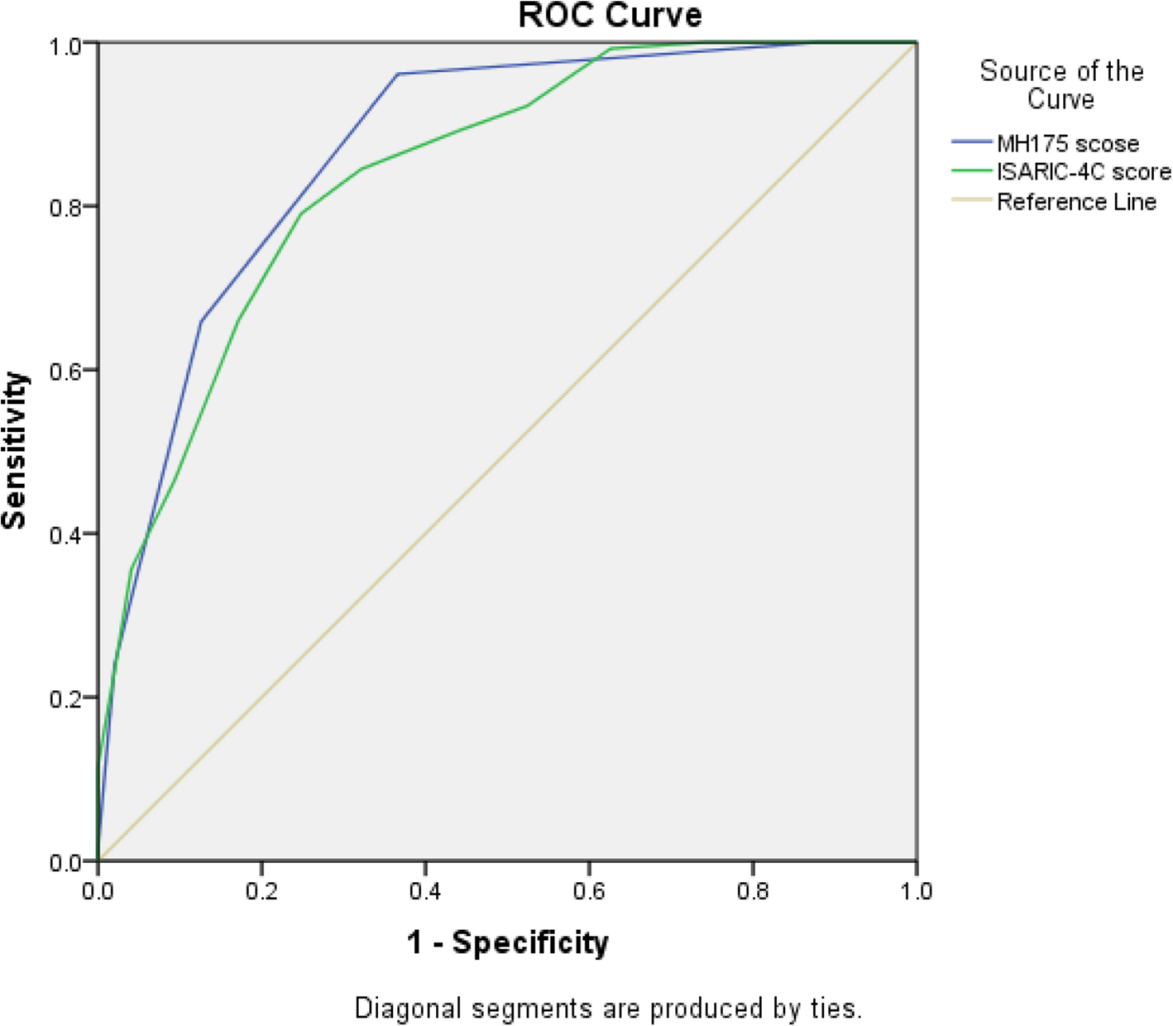
The ROC curves of the MH175 score and ISARIC-4C score

 The area under the receiver operating characteristic (AUROC) curve for the MH175 scale was 0.869 (95% CI, 0.83–0.9), indicating good discriminative ability. The optimal threshold value for predicting mortality using the MH175 score was determined to be ≥ 3 points. In comparison, the AUROC for the ISARIC-4C scale was 0.841 (95% CI, 0.8–0.88) (Fig. 2). When considering a threshold of MH175 ≥ 3 points, the scale exhibited a sensitivity of 96.1%, specificity of 63.4%, positive predictive value of 58%, and negative predictive value of 96.9% in COVID-19 patients. (Table 5).

**Table 5:** Accuracy of the MH175 score in estimating the risk of mortality

MH175		Mortality *(n* = 129)	Survival (*n* = 246)	Ss (%)	Sp (%)	PPV	NPV
Score	≥ 3	124 (96.1%)	90 (36.6%)	96.1	63.4	58	96.9
	< 3	05 (3.9%)	156 (63.4%)				

## Discussion

The clinical presentation of COVID-19 is highly diverse, and its rapid progression poses challenges for prognosis and classification in clinical settings. Our study revealed that age over 50, a requirement for prehospital oxygen therapy, and the presence of multiple comorbidities were closely associated with an increased risk of mortality. These findings were consistent with several other published studies [6–8].

 Symptoms such as fever, cough, and shortness of breath are commonly observed in patients and typically manifest soon after viral infection of the respiratory system. The severity of respiratory symptoms, ranging from dyspnea to acute respiratory failure, depends on the extent of lung damage. Acute respiratory failure and progressive multiorgan damage may lead to acute disturbances of consciousness. Our study identified fever, dyspnea, acute disturbances of consciousness, blood oxygen saturation below 94% (SpO2 < 94%), and the immediate need for oxygen support upon admission as factors significantly associated with the risk of mortality. These findings align with reports from other authors who have highlighted the prognostic significance of advanced age, dyspnea, impaired consciousness, and hypoxemia in predicting disease progression and mortality in hospitalized COVID-19 patients [9–11].

 Since the early stages of the pandemic, numerous studies have focused on developing prognostic models to stratify patients effectively and reduce the rates of severe disease progression and mortality. In a review conducted by C. Buttia et al. (2022), a total of 314 published studies from 40 countries were identified, including 152 studies on mortality prognosis and 35 studies on mortality prognosis and intensive resuscitation treatment. Factors found to have a significant impact on mortality risk included age, sex, hypoxemia, body temperature, pulse, underlying diseases, impaired consciousness, levels of C-reactive protein (CRP), urea, and D-dimer, neutrophil count, lymphocyte percentage, and platelet count. The area under the receiver operating characteristic (ROC) curve for mortality prognostic models ranged from 0.49 to 0.99, with sensitivity ranging from 15.4% to 100% and specificity ranging from 10.9% to 98.7% [12].

 With the aim of developing a clinical symptom-based prognostic tool for early risk classification upon hospital admission, we focused on selecting objective and easily assessable clinical factors associated with mortality risk. Eight clinical factors were identified from the univariate analysis as simple, objective, and significantly associated with mortality risk. These factors were further analyzed using multivariate analysis, revealing age over 50 years, multiple comorbidities, dyspnea, acute disturbance of consciousness, SpO2 below 94%, and oxygen demand exceeding 5 L per minute upon admission as independent predictors of mortality. A logistic regression algorithm was applied to construct a predictive model for mortality in COVID-19 patients, demonstrating favorable performance across all evaluation measures. This model facilitates risk stratification of patients and has the potential to reduce mortality rates within hospitals [13]. Prognostic models generated through multivariate analysis hold significant value, particularly in individualized predictions, by providing insights into a patient's likelihood of survival [14]. In a previous study, we utilized the multivariate analysis approach to assess the prognostic capability of the CURB-65 scale, which comprises five variables (consciousness disorder, uremia, tachypnea, low blood pressure, and age ≥ 65 years), for mortality prediction in hospitalized pneumonia patients with COVID-19. Our findings indicated that the CURB-65 scale exhibits promising prognostic ability, with a CURB-65 score of ≥ 2 points demonstrating a sensitivity of 82% and specificity of 83% in predicting mortality [15].

 The findings from our study demonstrated a proportional increase in the mortality rate and MH175 score. When the MH175 score reached ≥ 3 points, the mortality rate exceeded 39.8%. The MH175 score exhibited a good prognostic capability for mortality, as indicated by an area under the AUROC of 0.87. The optimal threshold value for predicting mortality using the MH175 score was ≥ 3 points, yielding a sensitivity of 96.1%, specificity of 63.4%, positive predictive value of 58%, and negative predictive value of 96.9%.

In a meta-analysis conducted to assess the predictive performance of four common prognostic scores (ISARIC-4C, COVID-GRAM, qCSI, and NEWS) for in-hospital mortality in COVID-19 patients, a favorable predictive value for mortality risk was observed. The ISARIC-4C score exhibited the highest AUROC at 0.799, followed by COVID-GRAM with 0.785, NEWS with 0.764, and qCSI with 0.749 [16].

 Although the complexity of the pandemic and variations in healthcare systems across different countries may introduce biases in prognostic models, the ISARIC-4C mortality prognostic model, developed based on a large database from the UK, has demonstrated a relatively low risk of bias. The 4C scale incorporates eight variables: age, sex, respiratory rate, oxygen saturation, comorbidities, state of consciousness, blood urea, and C-reactive protein [6, 17]. The clinical variables included in the MH175 scale were comparable to those in the 4C scale. However, a major distinguishing feature of the MH175 scale is that its variables can be promptly and easily assessed upon patient admission to the hospital, rendering the MH175 scale practical and feasible for prognosis and patient classification in primary healthcare settings.

 Currently, the COVID-19 pandemic continues to pose a significant threat to human health despite efforts to control its spread. Mortality rates remain high, particularly among elderly patients who are immunocompromised or have many comorbidities. Additionally, the potential for new strains and outbreaks adds to the ongoing risk. To address these challenges, our study proposes an additional tool for the early prediction of mortality risk in COVID-19 patients. This tool aims to facilitate efficient patient stratification, improve treatment efficacy, and ultimately reduce mortality.

 However, it is important to acknowledge the limitations of our study. First, as a single-center study, the generalizability of the findings may be limited. Second, the sample size used in the study was relatively small, necessitating further investigation with larger sample sizes. Moreover, the study's limitation lies in assigning a score to each clinical variable without precisely assessing its clinical significance. Additionally, regarding the MH175 score's prognostic ability for risks such as HFNC ventilation, NIV, and ICU admission, we did not conduct further analysis in this study, which we acknowledge as an additional limitation. Last, in the context of a pandemic, there is a lack of consensus regarding the initial diagnosis and treatment protocols for patients prior to admission, which could potentially influence the risk of mortality.

 It is crucial to recognize that all prognostic models have inherent limitations when applied in clinical practice, particularly in the context of a pandemic with diverse patient characteristics across different countries and races, as well as variations in healthcare systems. Ideally, highly effective prognostic models should undergo rigorous evaluation across various populations and countries before being widely implemented in clinical practice. This approach helps mitigate errors in decision-making, patient classification, and treatment choices [18].

## Conclusions

Based on our study involving 375 hospitalized COVID-19 patients at Military Hospital 175, we have drawn several key findings. First, the MH175 scale exhibits a favorable ability to predict the risk of mortality, as indicated by its area under the receiver operating characteristic (AUROC) value of 0.87. Additionally, there is a direct correlation between the MH175 score and mortality, with an increasing score corresponding to an elevated mortality rate. Moreover, a threshold value of ≥ 3 points on the MH175 scale emerges as an effective indicator for predicting mortality risk, demonstrating a sensitivity of 96.1%, specificity of 63.4%, positive predictive value of 58%, and negative predictive value of 96.9%. These promising results suggest that the MH175 scale can be implemented in clinical settings to enable early identification of mortality risk, facilitate patient stratification, and aid in selecting appropriate treatment interventions, ultimately reducing the likelihood of death among COVID-19 patients. Additionally, the score scale can serve as a tool to assist in classifying patients based on their risk levels, facilitating appropriate patient allocation within the treatment hierarchy.

## Data Availability

The data supporting the findings of this study are available from the corresponding author upon reasonable request. The data were not publicly available because of privacy and ethical restrictions.

## Abbreviations

COVID-19: Coronavirus Disease 2019
MH175: Military Hospital 175
WHO: World Health Organization
ARDS: Severe acute respiratory distress syndrome
SARS-CoV-2: Severe acute respiratory syndrome coronavirus 2
Saturation of Peripheral Oxygen: SpO2
RT‒PCR: Reverse transcription polymerase chain reaction
ROC: The receiver operating characteristic
AUROC: The area under the receiver operating characteristic curve
